# Machine Learning Based on Routine Hematological Parameters and Derived Inflammatory Indices for the Diagnosis of Schizophrenia

**DOI:** 10.3390/biomedicines14102209

**Published:** 2026-09-29

**Authors:** Xiaomei Fu, Weifeng Jin, Dan Li, Zhenhua Li, Qing Chen, Shuzi Chen, Peijun Ma, Mengyuan Zhu, Mengxia Wang, Caiwei Qu, Ruoxuan Pan, Zhiyun Chai, Ping Lin

**Affiliations:** Department of Medical Laboratory, Shanghai Mental Health Center, Shanghai Jiao Tong University School of Medicine, Shanghai 200030, China; fuxiaomei202010@163.com (X.F.);

**Keywords:** schizophrenia, routine hematological parameters, machine learning, major depressive disorder

## Abstract

**Background**: Schizophrenia is frequently underdiagnosed or diagnosed late due to the lack of objective diagnostic markers. This study aimed to develop and validate a machine learning model using routine hematological parameters for the auxiliary diagnosis of schizophrenia. **Methods**: A total of 150 first-episode drug-naïve schizophrenia patients and 150 healthy controls were enrolled. Study parameters included routine hematological parameters and its derived inflammatory markers. Feature selection was performed using Elastic Net regression, followed by the construction of an L2-regularized logistic regression model. Model discriminative performance, calibration, and clinical utility were assessed through internal validation, area under the receiver operating characteristic curve (AUC), calibration curves, and decision curve analysis. An independent cohort of 50 schizophrenia patients and 50 patients with major depressive disorder (MDD) was used for exploratory differential diagnostic evaluation. **Results**: The final model incorporated 12 features: neutrophil count (NEUT), eosinophil count (EO), mean platelet volume (MPV), hematocrit (HCT), hemoglobin (HGB), neutrophil-to-lymphocyte ratio (NLR), lymphocyte-to-monocyte ratio (LMR), systemic immune-inflammation index (SII), systemic inflammation response index (SIRI), neutrophil-to-platelet ratio (NPR), along with age and sex. The model achieved an AUC of 0.859 (95% CI: 0.780–0.937) on the test set, with an accuracy of 0.767, sensitivity of 0.711, and specificity of 0.822. Calibration curves confirmed good calibration, and decision curve analysis further verified its clinical utility. In the exploratory differential diagnostic analysis, the model showed limited performance in distinguishing schizophrenia from MDD. **Conclusions**: The model based on routine hematological parameters and L2-regularized logistic regression can effectively differentiate first-episode drug-naïve schizophrenia patients from healthy controls, providing a low-cost and easily accessible auxiliary diagnostic tool for clinical practice.

## 1. Introduction

Schizophrenia (SCZ) is a severe and chronic mental disorder, affecting approximately 24 million individuals worldwide [1]. Most patients experience persistent cognitive impairment as well as affective and psychomotor disturbances throughout the course of the illness, which profoundly impair daily functioning and social performance [2]. At present, the clinical diagnosis of SCZ still relies primarily on structured clinical interviews and behavioral observation, and no biomarkers have yet been incorporated into current clinical diagnostic guidelines as established criteria. Although the exact etiology and pathogenesis of the disorder remain incompletely understood, a growing body of evidence has demonstrated that systemic immune dysfunction, along with immunoinflammatory processes within the central nervous system, plays a critical role in the onset and progression of SCZ [3]. Over the years, researchers have devoted considerable effort to elucidating the pathogenesis of SCZ and to identifying biomarkers that could facilitate objective diagnosis of the disorder.

In recent years, the role of systemic inflammation in the pathophysiology of SCZ has received increasing attention [4,5]. Accumulating evidence has demonstrated immune abnormalities in the peripheral blood, cerebrospinal fluid, and central nervous system of patients with SCZ, manifested as alterations in immune cell counts, elevated inflammatory markers, and exacerbated oxidative stress [6,7,8]. Routine hematological parameters, as a routine clinical laboratory test, provide a convenient reflection of peripheral blood cell composition and systemic inflammatory status. Multiple studies have consistently reported that peripheral neutrophil counts and the neutrophil-to-lymphocyte ratio (NLR) are significantly higher in patients with SCZ than in healthy controls [9,10,11]. Composite inflammatory indices such as the systemic immune-inflammation index (SII) and the systemic inflammation response index (SIRI) have also been found to be associated with disease status in SCZ [12]. These findings suggest that cell counts and their derived ratios from routine hematological testing hold translational potential as auxiliary diagnostic tools for SCZ.

In recent years, the growing availability of large-scale datasets has substantially promoted the application of machine learning (ML) techniques in psychiatric research. Compared with traditional statistical methods, machine learning excels at handling high-dimensional data and capturing complex non linear relationships among variables, and has demonstrated favorable performance in distinguishing patients from healthy controls using diverse data types [13,14,15]. Several studies have applied machine learning to the diagnosis of SCZ: Yee et al. developed a classification model based on 18 plasma inflammatory proteins, achieving an area under the receiver operating characteristic curve (AUC) of 0.74 [16]. Varathan et al. constructed a classification model integrating transcriptomic, miRNA, and epigenomic data, with an AUC reaching 0.86 [17]. These studies have preliminarily validated the feasibility of combining machine learning with peripheral biomarkers for disease discrimination.

The primary objective of this study is to explore the value of machine learning based classification models using routine hematological parameters for the auxiliary diagnosis of SCZ. Specifically, we sought to develop a classification model using L2-regularized logistic regression, evaluate its discriminative performance and generalizability in distinguishing patients with SCZ from healthy controls, and thereby provide a low-cost, easily accessible auxiliary decision-making tool for clinical practice. In addition, we preliminarily explored the model’s differential diagnostic utility between SCZ and major depressive disorder (MDD) in an independent dataset.

## 2. Materials and Methods

### 2.1. Participants

**Figure 1:**
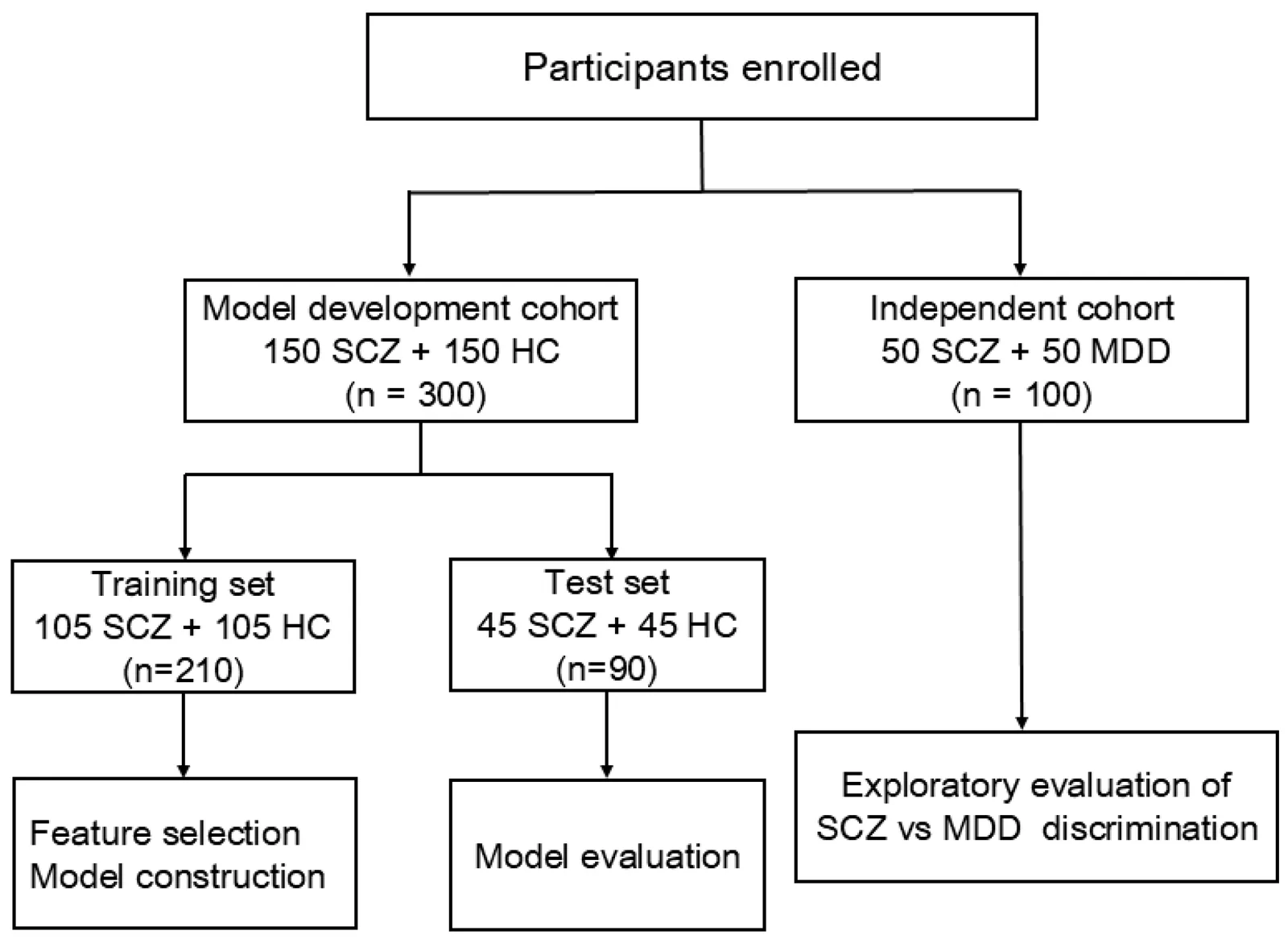
Study flow diagram. A total of 300 participants were enrolled in the model development cohort and stratified into a training set and a test set at a 7:3 ratio. A separate independent cohort was used for the exploratory differential-diagnostic analysis.

First-episode patients with SCZ or MDD were enrolled at Shanghai Mental Health Center, and healthy controls were recruited from staff attending their routine annual physical examination at the same center. A total of 150 SCZ patients and 150 healthy controls (HC) were enrolled for model development. An independent dataset comprising 50 SCZ patients and 50 MDD patients was used to evaluate the specificity of the model. The study was approved by the Ethics Committee of Shanghai Mental Health Center (2024-70), and written informed consent was obtained from all participants or their legal guardians. Figure 1 illustrates the research flowchart.

### 2.2. Inclusion and Exclusion Criteria

Inclusion criteria for the SCZ group were as follows: (1) diagnosis of schizophrenia according to the Diagnostic and Statistical Manual of Mental Disorders, Fifth Edition (DSM-5) criteria by two attending psychiatrists, (2) age 18–65 years, and (3) first-episode and antipsychotic-naïve at enrollment. Exclusion criteria for the SCZ group included: (1) comorbid psychiatric disorders or severe physical illnesses, (2) acute or chronic infections, autoimmune diseases, hematological disorders, or malignancies, (3) pregnancy or lactation, and (4) blood transfusion or use of immunomodulatory agents within the preceding 3 months. For HC, the absence of personal or family history of psychiatric disorders was confirmed by direct inquiry, and they were subject to the same exclusion criteria as the SCZ group. The inclusion and exclusion criteria for the MDD group were identical to those for the SCZ group, except that the diagnosis was major depressive disorder according to DSM-5 and patients were antidepressant-naïve at enrollment.

### 2.3. Specimen Analysis

Fasting venous blood (2 mL) was drawn from patients prior to treatment and from healthy controls at enrollment. Complete blood count parameters were measured using a Sysmex XN-20 automated analyzer (Sysmex Corporation, Kobe, Japan).

### 2.4. Feature Selection and Data Preprocessing

Given that age and sex are closely associated with the onset of SCZ, these two factors were included in the model. A total of 26 features were included: (1) sex and age, (2) laboratory parameters, including white blood cell count (WBC), neutrophil count (NEUT), lymphocyte count (LYMPH), monocyte count (MONO), eosinophil count (EO), basophil count (BASO), red blood cell count (RBC), hemoglobin (HGB), hematocrit (HCT), mean corpuscular volume (MCV), mean corpuscular hemoglobin (MCH), mean corpuscular hemoglobin concentration (MCHC), red blood cell distribution width-coefficient of variation (RDW-CV), platelet count (PLT), mean platelet volume (MPV), and plateletcrit (PCT), and (3) derived inflammatory markers: NLR, platelet-to-lymphocyte ratio (PLR), lymphocyte-to-monocyte ratio (LMR), SII, SIRI, neutrophil-to-platelet ratio (NPR), eosinophil-to-lymphocyte ratio (ELR) and basophil-to-lymphocyte ratio (BLR). The derived markers were calculated using the following formulas: NLR = NEUT/LYMPH, PLR = PLT/LYMPH, LMR = LYMPH/MONO, SII = (NEUT × PLT)/LYMPH, SIRI = (NEUT × MONO)/LYMPH, NPR = NEUT/PLT, ELR = EO/LYMPH, and BLR = BASO/LYMPH.

The model development set was stratified split into training and test sets at a ratio of 7:3, and all screening operations were performed exclusively within the training set. Outliers were identified using the interquartile range (IQR) method and winsorized. Age was standardized using Z-score normalization. Because the subsequently used models (L1/L2-regularized logistic regression and linear kernel support vector machine (SVM)) are sensitive to feature magnitude, the other continuous variables were scaled using RobustScaler.

Age and sex were forced into the model as covariates. Feature selection was performed on the 24 candidate variables in the training set (*n* = 210; 105 SCZ and 105 HC) using a two-step residual approach combined with Elastic Net regression [18]. The regularization parameter (λ) was determined via ten-fold cross-validation. Both the λ value corresponding to the minimum cross-validated error (λ_min) and the largest λ within one standard error of the minimum (λ_1se) were computed. The optimal λ was selected based on the one-standard-error rule, aiming to obtain a more parsimonious model with minimal loss in predictive performance.

### 2.5. Machine Learning

L2-regularized logistic regression was adopted as the primary model, using the final feature set as predictors and SCZ diagnosis as the outcome. The regularization parameter was optimized automatically via cross-validation. Ridge logistic regression and SVM were also trained as complementary models. Since ridge logistic regression is mathematically equivalent to the L2-regularized model, it additionally served as an internal consistency check of our pipeline. For coefficient inference in the L2-regularized logistic model, conventional standard errors based on the Fisher information matrix and Wald test *p*-values are not theoretically justified due to the bias introduced by regularization. Therefore, bootstrap resampling with 1000 iterations was performed to estimate the empirical distribution of each coefficient, and 95% confidence intervals were derived using the percentile method to evaluate the robustness of the associations.

To assess overfitting, cross-validation was conducted within the training set and learning curves were generated. Model discrimination and calibration were evaluated on the independent test set. To examine the robustness of generalization performance, the 7:3 stratified splitting procedure was repeated 500 times. The AUC was recorded for each test set, and the mean AUC with its confidence interval was computed. Additionally, decision curve analysis was employed to assess the clinical net benefit of the model.

Furthermore, an independent dataset (50 SCZ and 50 MDD patients) was applied to the models for an exploratory analysis of differential diagnostic specificity.

### 2.6. Statistical Analyses

For baseline characteristics, categorical variables were presented as percentages and compared between groups using the chi-square test. For continuous variables, normality was first assessed. Normally distributed continuous variables were expressed as mean ± standard deviation (SD) and compared using the independent samples *t*-test. Non-normally distributed continuous variables were presented as median with IQR and compared using the Mann–Whitney U test. A two-tailed *p* value < 0.05 was considered statistically significant. All statistical analyses and machine learning procedures were performed using Python (version 3.13.0). A fixed random seed of 42 was set to ensure reproducibility.

## 3. Results

### 3.1. Baseline Characteristics

A total of 210 participants were included in the training set, consisting of 105 SCZ patients and 105 HCs. The two groups were well matched for sex and age (*p* = 0.619), with no significant between-group differences (Table 1). Among the routine hematological parameters, significant differences between the SCZ and HC groups were observed for NEUT, MONO, EO, MPV, LYMPH, WBC, HCT, and HGB (all *p* < 0.05). Furthermore, all eight derived markers showed significant differences between the two groups (Table 1). The baseline characteristics of the training set were comparable to those of the overall cohort (*n* = 300) (Table A1).

**Figure 2:**
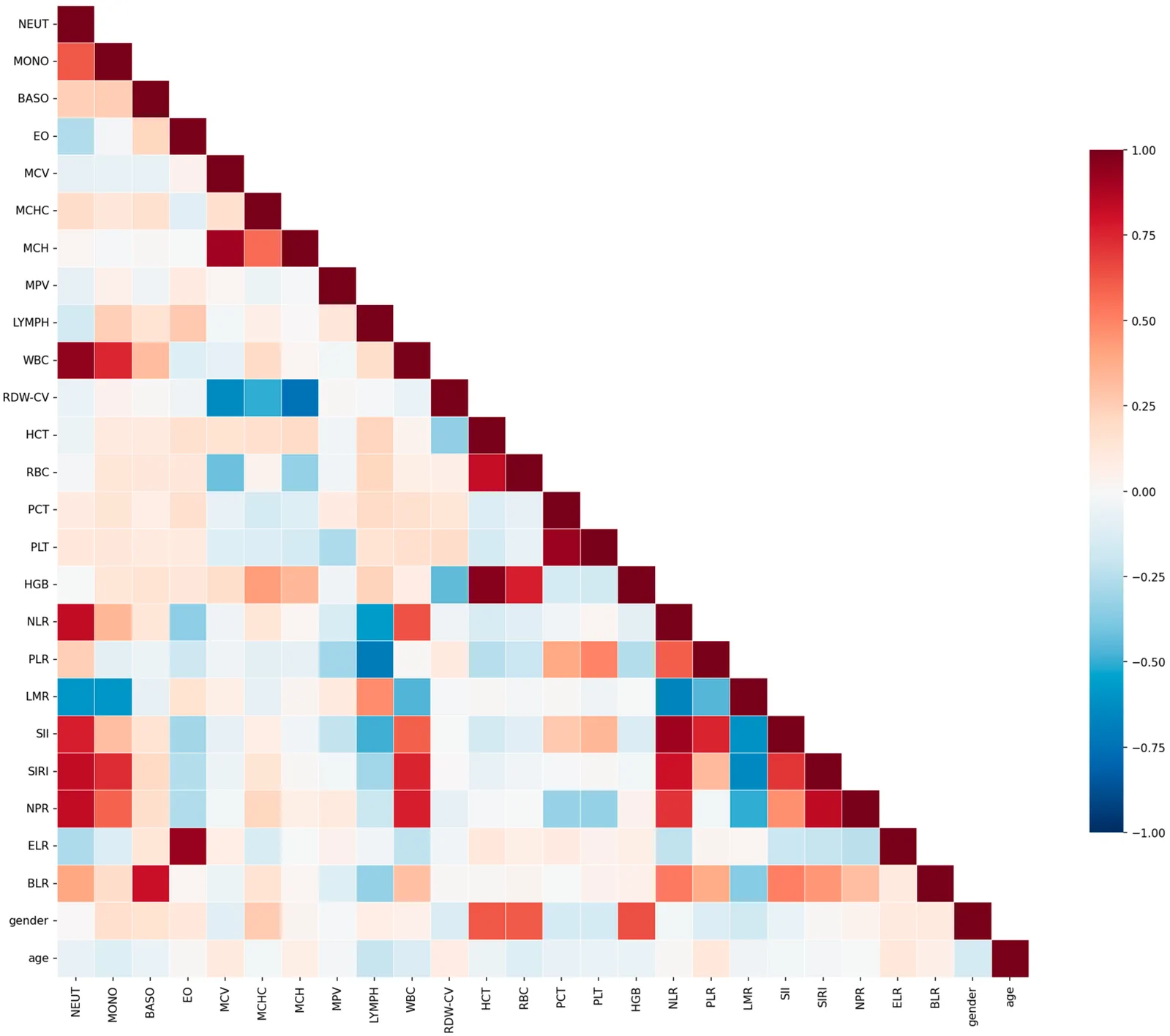
Correlation coefficient heatmap of the 26 candidate variables.

To explore the correlational structure among the candidate variables, a correlation coefficient heatmap of the 26 variables was plotted (Figure 2). The results revealed high multicollinearity between individual hematological parameters and their derived markers, as well as moderate-to-strong correlations among the derived markers themselves. In particular, HGB and HCT were strongly correlated (r = 0.96), and the derived indices NLR, SII, SIRI, and NPR were highly correlated with NEUT (r = 0.84, 0.77, 0.84, and 0.83, respectively). Therefore, Elastic Net regression was subsequently employed for feature selection. Elastic Net performs coefficient shrinkage and variable selection that is robust to multicollinearity [18].

### 3.2. Variables Selection Results

**Figure 3:**
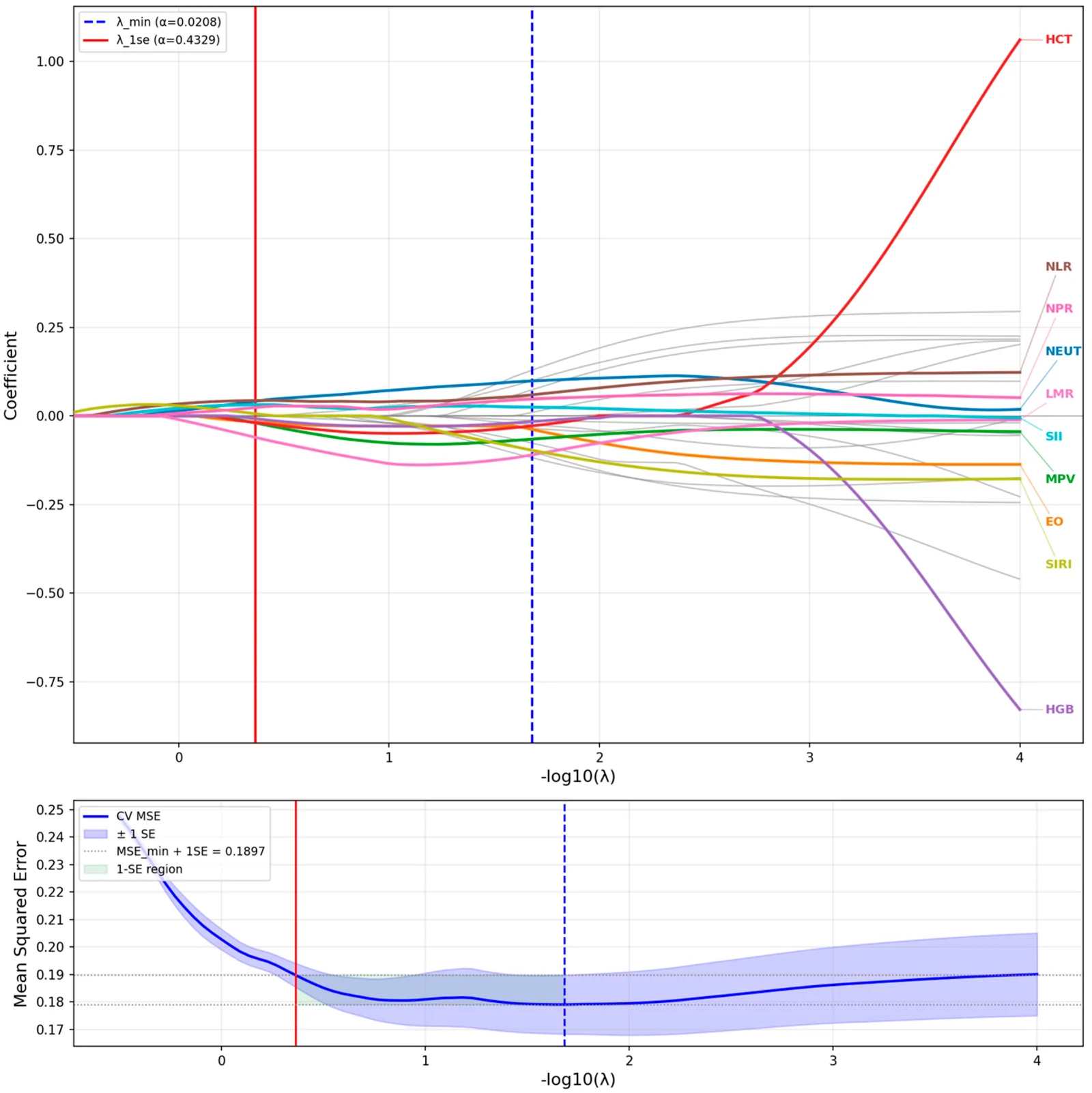
Elastic Net coefficient path plot and 10-fold cross-validation mean squared error (MSE) curves. Upper panel: Coefficient shrinkage paths of the 24 candidate hematological variables; the 10 variables selected by Elastic Net under λ_1se are shown in bold colors and labeled at the end of their paths, while the remaining 14 non-selected variables are shown in light gray. Lower panel: Ten-fold cross-validation MSE curves with ±1 SE bands. λ_1se was selected under the 1-SE rule, yielding 10 variables with non-zero coefficients.

Based on the two regularization parameters, λ_min (0.021) and λ_1se (0.433), the model retained 21 and 10 variables, respectively. To achieve a more parsimonious model with minimal loss in predictive performance, the model corresponding to λ_1se was ultimately selected, yielding 10 variables (Figure 3). Among these, five were routine hematological parameters (NEUT, EO, MPV, HCT, HGB) and five were derived inflammatory markers (NLR, LMR, SII, SIRI, NPR). Consistent with the grouping effect of Elastic Net, some of the retained variables were mutually correlated.

### 3.3. Training Set Model Performance

The 10 variables selected by Elastic Net, together with age and sex, were entered into an L2-regularized logistic regression model, giving 12 predictors in total (optimal C = 0.117). Bootstrap-derived coefficients and odds ratios (ORs) with 95% confidence intervals (CIs) for this final model are presented in Table 2. Among the selected variables, five had positive coefficients: NEUT, NLR, SII, SIRI, and NPR. Four had negative coefficients: MPV, HCT, HGB, and LMR. The coefficient of EO was also negative, but its 95% CI crossed the null, indicating that its independent contribution was not robust. Age and sex served solely as adjustment covariates and were not interpreted as biomarkers.

A 10-fold cross-validation was performed on the training set. The primary model (L2-regularized logistic regression) achieved a mean AUC of 0.844 (SD = 0.099), with an accuracy of 0.814, sensitivity of 0.745, specificity of 0.886, and Brier score of 0.156 (Table 3). Ridge logistic regression, being mathematically equivalent to the primary model, yielded identical performance metrics. The linear kernel SVM achieved an AUC of 0.841 (SD = 0.104), which was comparable to that of the primary model; however, it exhibited lower sensitivity (0.564) and higher specificity (0.963). The linear kernel SVM was inferior to the primary model in balancing sensitivity and specificity (Table 3).

**Figure 4:**
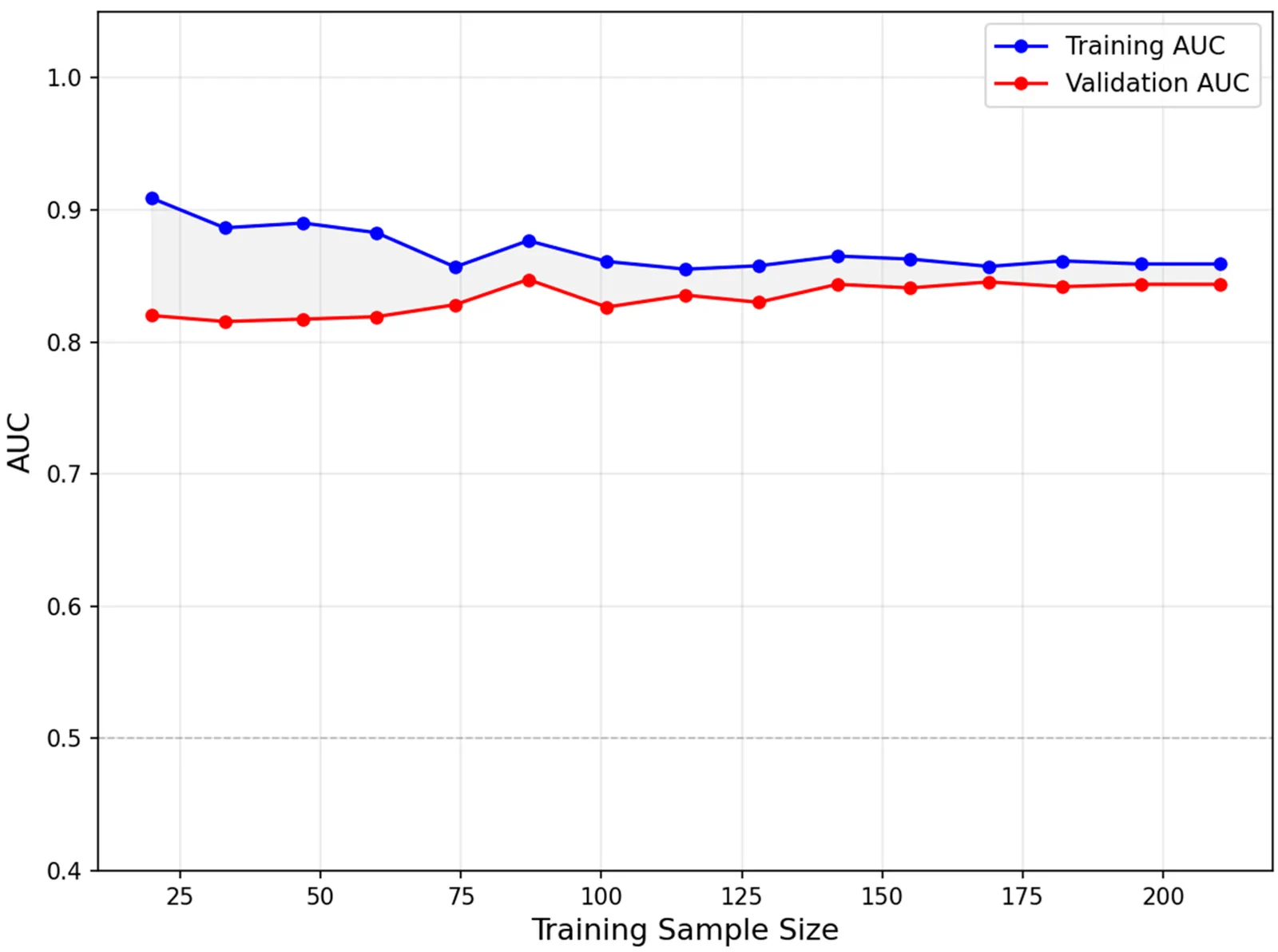
Learning curves of the primary model on the training set.

The learning curves (Figure 4) showed that the AUCs of the training and validation sets gradually converged as the training sample size increased, with the AUC gap narrowing from an initial 0.049 to approximately 0.015 and remaining stable thereafter, without a diverging trend, indicating a low risk of overfitting.

### 3.4. Test Set Evaluation

#### 3.4.1. Comparative Discriminative Performance of Models

**Figure 5:**
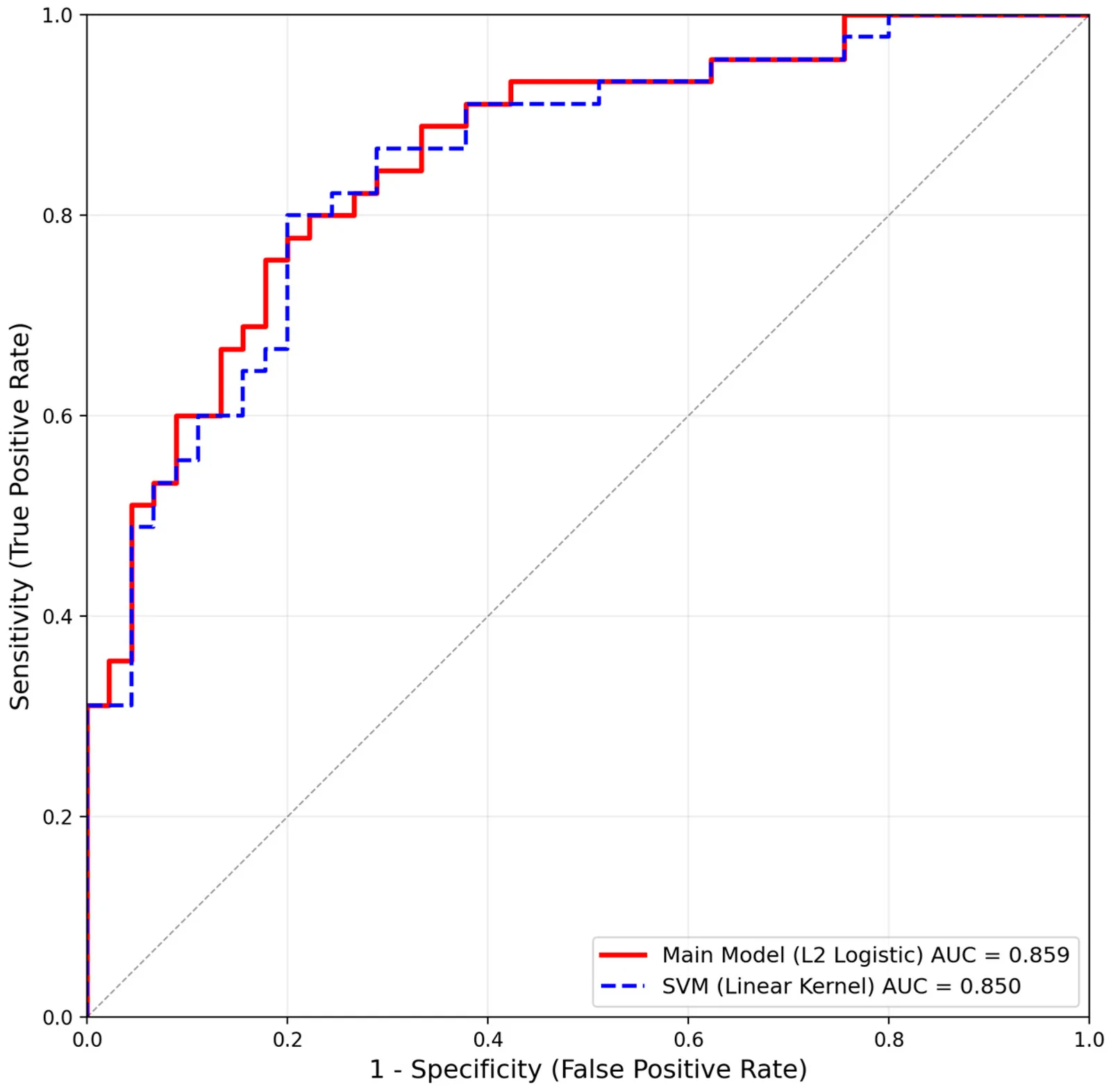
Receiver operating characteristic (ROC) curves of the models on the test set.

Performance metrics for each model on the test set are summarized in Table 4, with corresponding ROC curves presented in Figure 5. The primary model achieved a test-set AUC of 0.859 (95% CI: 0.780–0.937), marginally surpassing the 10-fold cross-validation mean of 0.844. Ridge logistic regression produced performance metrics identical to those of the primary model, as the two models are mathematically equivalent. The linear kernel SVM yielded an AUC of 0.850 (95% CI: 0.769–0.931), which was slightly inferior to that of the primary model.

#### 3.4.2. Model Calibration

**Figure 6:**
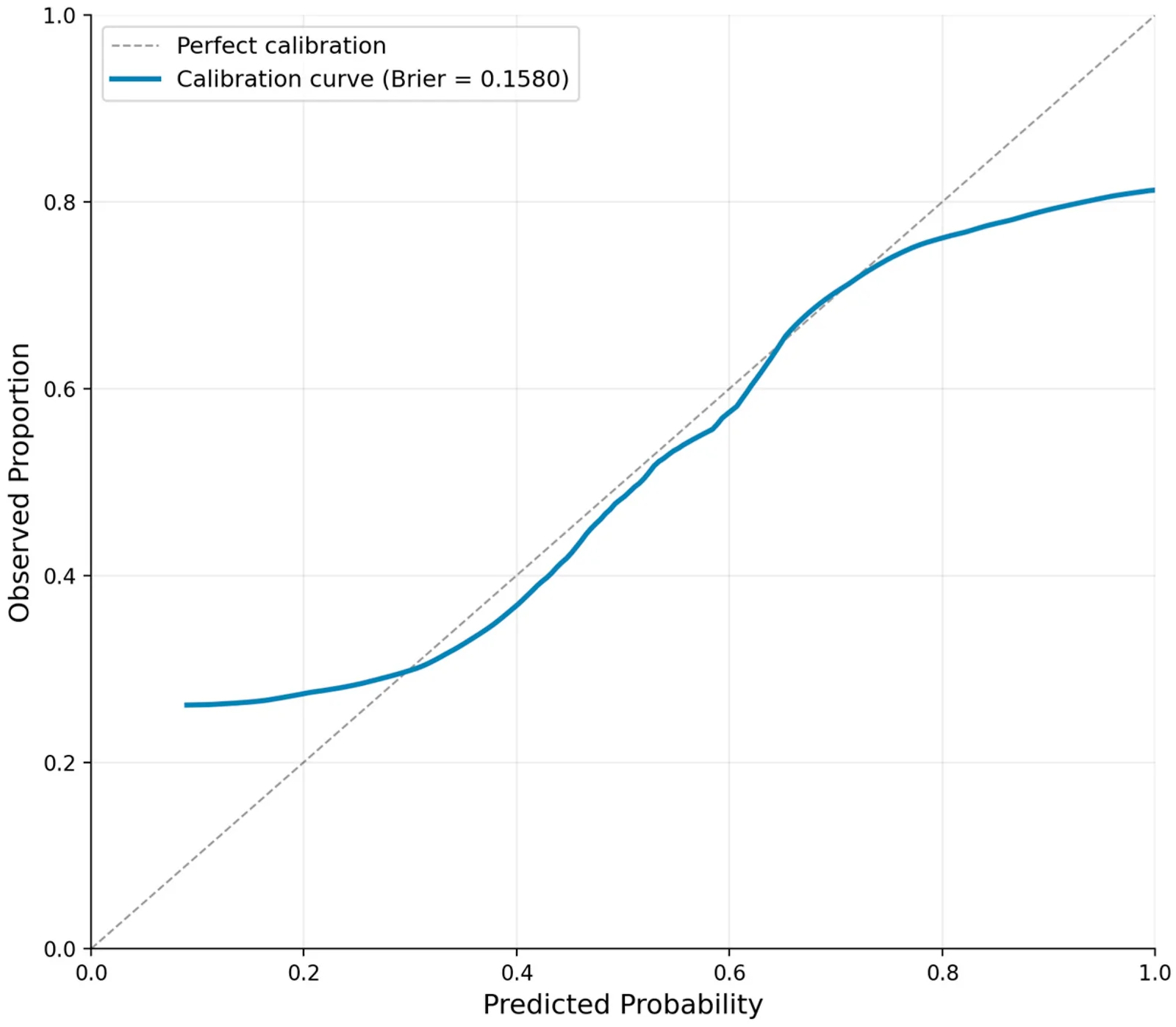
Calibration curve of the primary model on the test set.

The calibration curve for the test set (Figure 6) demonstrated generally good agreement between predicted probabilities and observed outcomes. The Brier score was 0.1580, indicating acceptable model calibration. This result was further corroborated by the Hosmer-Lemeshow goodness-of-fit test (χ^2^ = 5.455, df = 8, *p* = 0.708).

#### 3.4.3. Stability of Generalization Performance

**Figure 7:**
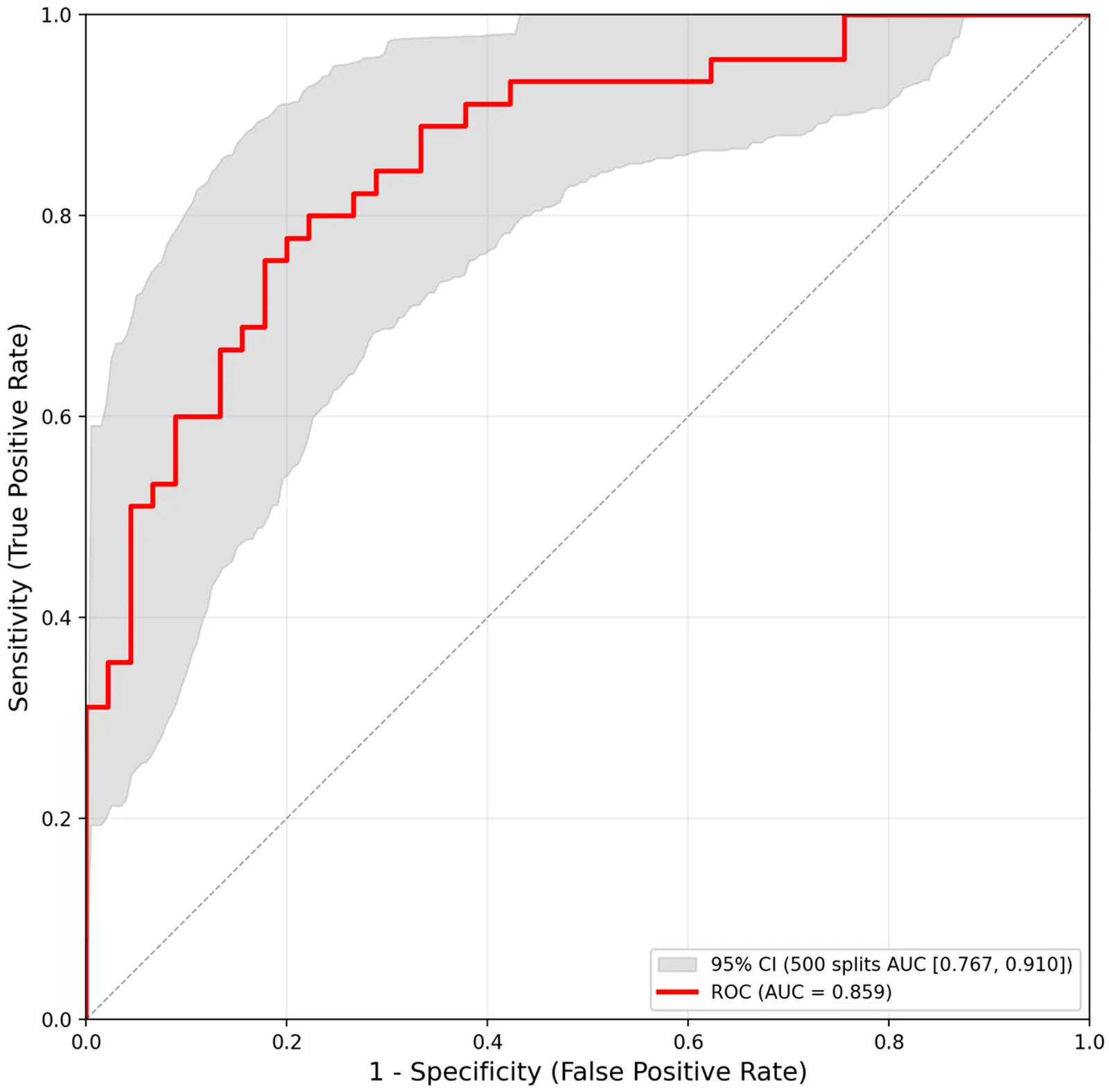
Bootstrap validation of the main model (500 iterations).

The stability of the model was evaluated using the Bootstrap method. Figure 7 presents the ROC curves generated from 500 bootstrap resampling iterations, all of which clustered closely around the ROC curve of the fixed test set. The mean AUC across bootstrap samples was 0.841 (SD = 0.037, 95% CI: 0.767–0.910), which was consistent with the fixed test-set AUC of 0.859, confirming that the primary model possessed favorable generalizability and stability.

#### 3.4.4. Clinical Utility

**Figure 8:**
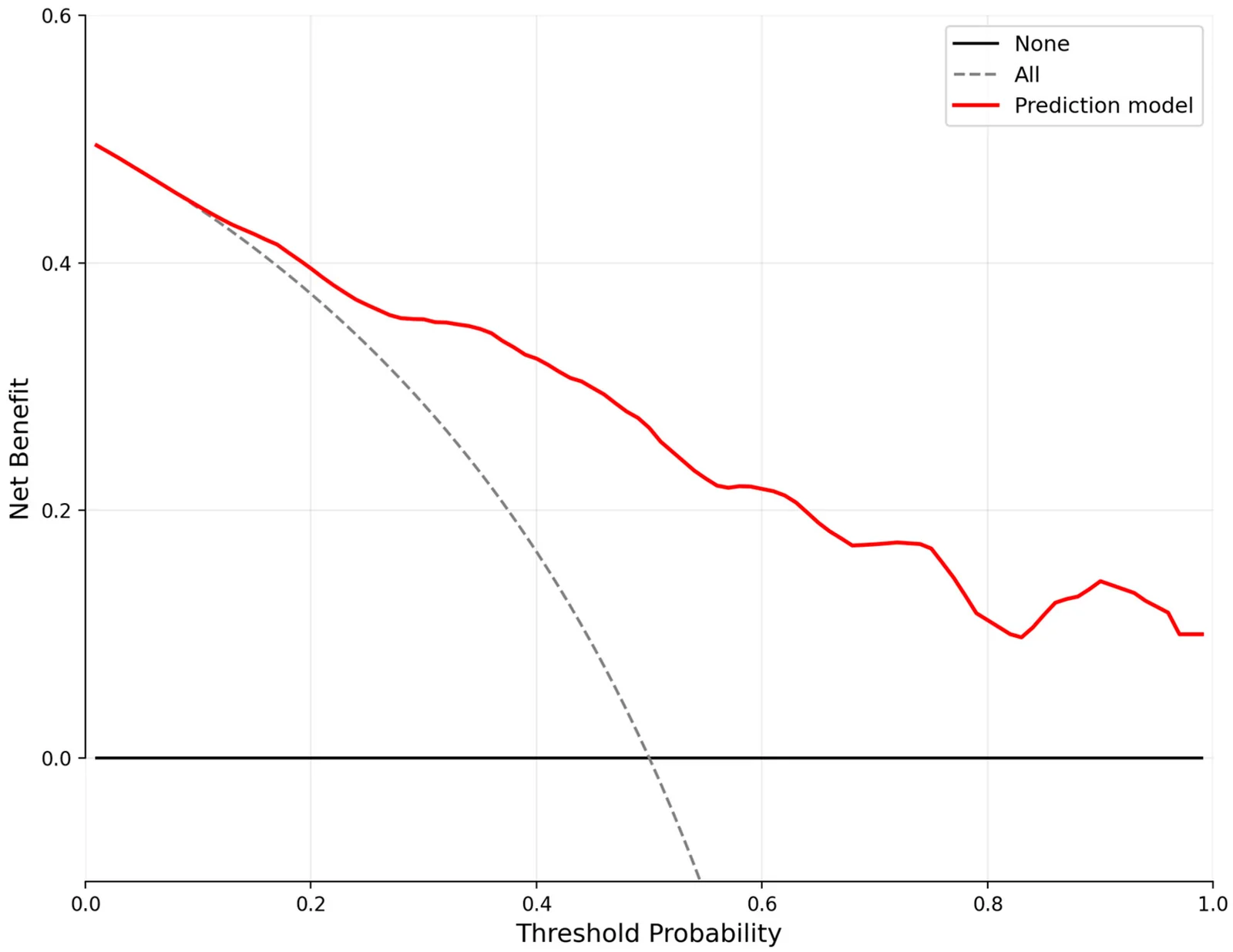
Decision curve analysis (DCA) of the primary model.

Decision curve analysis (Figure 8) demonstrated that the primary model provided a significant net benefit for identifying patients with SCZ across a wide range of risk threshold probabilities (10–100%) in the test set.

### 3.5. Exploratory Differential Diagnostic Analysis

To preliminarily investigate the potential value of the inflammatory marker-based model in psychiatric differential diagnosis, the primary model was directly applied to an independent dataset (*n* = 100, 50 SCZ and 50 MDD). The results showed that the model AUC decreased to 0.613, which was substantially lower than its performance in discriminating SCZ from HC (0.859). At the optimal threshold, the model yielded an accuracy of 60%, a sensitivity of 76%, and a specificity of only 44% for SCZ identification.

## 4. Discussion

In this study, we developed an auxiliary diagnostic model for SCZ using routine hematological parameters. Feature selection was performed via Elastic Net regularization, and a diagnostic model was constructed using L2-regularized logistic regression. The model achieved a test-set AUC of 0.859 (95% CI: 0.780–0.937). The ten features selected by Elastic Net—NEUT, EO, MPV, HCT, HGB, NLR, LMR, SII, SIRI, and NPR—span multiple blood cell lineages, including neutrophils, lymphocytes, monocytes, platelets, and erythrocytes. This finding suggests that peripheral immune dysregulation in SCZ exhibits a multi-lineage, systemic pattern, rather than being confined to a single leukocyte subset.

In the feature selection stage, NEUT, NLR, SII, SIRI, and NPR all entered the final model with positive coefficients, and their effect directions were consistently retained in the subsequent L2-regularized logistic regression, with all bootstrap 95% confidence intervals not crossing the null, suggesting that elevated levels of these markers were stably associated with an increased risk of SCZ. A systematic review reported that peripheral neutrophil counts were significantly higher in patients with first-episode psychosis and SCZ compared with healthy controls, and this difference was independent of antipsychotic medication use [11]. NLR exhibited a relatively large standardized coefficient in the present screening, with an effect size (OR = 1.433) ranking among the top across multiple markers. Previous studies have shown that peripheral NLR is significantly elevated in patients with SCZ compared with healthy controls [7,10]. Elevated NLR generally indicates a chronic low-grade inflammatory state, characterized by increased neutrophil counts and decreased lymphocyte counts. Sustained inflammation may contribute to the pathophysiology of SCZ through neuroinflammatory pathways and disturbances in neurotransmitter metabolism [19].

NPR demonstrated a relatively large effect size (standardized OR = 1.595) in the final model, ranking first among all selected variables. However, studies on the neutrophil-to-platelet ratio in SCZ patients remain limited. Of note, a recent study reported that PNR (platelet-to-neutrophil ratio) was significantly lower in first-episode drug-naïve SCZ patients than in healthy controls, and PNR emerged as one of the core predictors of suicidal ideation in this population [20]. This finding indirectly supports the potential significance of the ratio between neutrophil and platelet counts in the inflammatory pathology of SCZ, warranting further investigation.

SII and SIRI, as composite inflammatory indices integrating neutrophils, lymphocytes, and platelets (or monocytes), were also retained in the final feature set. Wu et al. found that both SIRI and SII were higher in SCZ inpatients with comorbid sleep disorders than in those without, suggesting that SII/SIRI may more closely reflect the inflammatory burden associated with comorbid conditions in SCZ [12]. In the present study, SII and SIRI were already significantly elevated in drug-naïve patients, indicating that the intrinsic immune abnormalities in SCZ are sufficient to drive the shifts in these composite markers, and comorbid conditions may further amplify this effect.

MPV, HCT, HGB, and LMR all entered the final model with negative coefficients, with their bootstrap 95% confidence intervals also not crossing the null, suggesting that decreased levels of these markers were stably associated with an increased risk of SCZ. MPV, an indirect indicator of platelet activation, showed a negative coefficient in the present study, which is consistent with the findings of Almış et al. [21]. However, evidence regarding the direction of MPV changes in SCZ remains inconsistent [22,23,24]. This discrepancy may be partially attributable to the differential effects of antipsychotic medications, as previous evidence has indicated that atypical antipsychotic use is an independent predictor of elevated MPV [25]. Thus, the direction of MPV changes may reflect the combined influence of disease state and pharmacological treatment on platelet function.

Erythrocyte-related parameters, including HCT and HGB, were also selected into the final feature set. A follow-up study of patients with SCZ reported a higher proportion of low HGB and low HCT at baseline, which is consistent with the findings of the present study [26]. The reduction in HGB and HCT may be closely linked to iron metabolism disturbances under chronic inflammatory conditions [27]. In addition, abnormal erythrocyte membrane phospholipid metabolism and microcirculatory disturbances have also been suggested as potential biological features of SCZ, although the relevant evidence is mainly derived from small-sample studies [28,29]. These findings suggest that the pathophysiological alterations in SCZ may not be confined to the immune system but may also involve a global shift in the hematopoietic system, meriting further study. The decrease in LMR may be associated with lymphocytopenia and monocyte activation, reflecting dysregulated immune modulation, which, together with elevated NLR, jointly points to a chronic low-grade inflammatory state from different perspectives [30].

The model developed in the present study achieved an AUC of 0.859, with a sensitivity of 0.711 and specificity of 0.822 on the test set. Although only age, sex, and routine hematological parameters were included in the modeling process, without incorporating other clinical or biochemical findings, the model still attained an accuracy of 0.77, suggesting that a limited set of features can effectively distinguish patients with SCZ from healthy controls. Compared with previous studies, the discriminative performance of the present model is at a relatively high level. Malashenkova et al. developed a classification model for SCZ using peripheral blood immunological markers, achieving an AUC of approximately 0.71 [31]. Gunasekara et al. constructed a classification model based on peripheral blood DNA methylation with an AUC of 0.86 [32]. However, its clinical application is constrained by high detection costs and technical complexity. In contrast, the present model utilizes routine hematological parameters as input features, which are cost-effective and operationally convenient. These advantages highlight its potential for clinical translation.

In the exploratory analysis distinguishing SCZ from MDD, the model’s AUC decreased to 0.613. Although the model maintained a relatively high sensitivity for SCZ (76%), its specificity was only 44%, indicating that more than half of the MDD patients were misclassified as SCZ. This result was somewhat expected, as patients with SCZ and MDD share more similarities with each other than either group does with healthy individuals, particularly in terms of overlapping inflammatory pathways. For instance, elevated NLR has been consistently reported in both SCZ and MDD [11]. Moreover, the training set in the present study comprised only SCZ patients and healthy controls, meaning that the model was trained solely on the decision boundary of “SCZ versus HCs” without any supervisory signal for direct SCZ versus MDD discrimination. The limited sample size of the MDD group (*n* = 50) may also have undermined the stability of the evaluation. Nevertheless, as an exploratory analysis, the present study provides preliminary evidence regarding the applicability boundaries of inflammatory markers in the differential diagnosis of psychiatric disorders.

Several limitations of this study should be acknowledged. First, the cross-sectional design only captures peripheral inflammatory status at the time of admission, precluding causal inferences between inflammatory markers and SCZ. Second, potential confounders such as dietary patterns, smoking status, and sleep quality could not be fully accounted for. Third, the data were derived from a single center with a relatively limited sample size, and although the model’s performance in distinguishing SCZ from HC was assessed through internal validation, no independent external cohort was included; external validation of the SCZ-versus-HC classification in multicenter, large-sample cohorts therefore remains an essential next step to confirm the model’s generalizability.

## 5. Conclusions

In this study, we constructed a robust diagnostic model for schizophrenia based on routine hematological parameters using Elastic Net–L2-regularized logistic regression; the model achieved an AUC of 0.859 (95% CI: 0.780–0.937), demonstrating good discriminative performance. Given that all input features are derived from clinical tests, the model offers good accessibility and holds considerable potential for application in primary healthcare settings. The markers retained in the mode revealed a state of peripheral immune dysregulation in schizophrenia, indicating broad disturbances in circulating immune cells that accompany the disorder. Future multicenter, longitudinal studies with independent external validation are warranted to confirm the model’s generalizability and to improve its differential diagnostic performance.

## Figures and Tables

**Table 1:** Baseline characteristics of the training set.

Variables	SCZ (*n* = 105)	HC (*n* = 105)	Test of Significance	*p*
Gender (female, %)	52 (49.5%)	52 (49.5%)	χ^2^ = 0.000	1
Age (years)	36.00 (27.00, 48.00)	36.00 (28.00, 47.00)	U = 5406.0	0.619
**R** **outine hematological parameters**				
NEUT (×10^9^/L)	5.03 (3.50, 7.13)	3.29 (2.64, 4.10)	U = 8101.0	<0.001
MONO (×10^9^/L)	0.52 (0.42, 0.63)	0.44 (0.35, 0.53)	U = 7460.0	<0.001
BASO (×10^9^/L)	0.03 (0.02, 0.04)	0.03 (0.02, 0.04)	U = 5432.0	0.676
EO (×10^9^/L)	0.06 (0.02, 0.13)	0.14 (0.08, 0.19)	U = 3317.0	<0.001
MCV (fL)	87.20 (85.10, 89.90)	88.20 (86.00, 91.00)	U = 4746.0	0.066
MCHC (g/L)	338.00 (332.00, 346.00)	338.00 (332.00, 345.00)	U = 5259.0	0.562
MCH (pg)	29.80 (28.70, 30.70)	30.00 (29.20, 31.10)	U = 5000.0	0.212
MPV (fL)	10.00 (9.40, 10.70)	10.40 (9.90, 11.00)	U = 4226.0	0.001
LYMPH (×10^9^/L)	1.77 (1.43, 2.29)	2.17 (1.85, 2.54)	U = 3879.0	<0.001
WBC (×10^9^/L)	7.72 (6.13, 9.67)	6.16 (5.25, 7.16)	U = 7935.0	<0.001
RDW-CV (%)	12.50 (12.10, 12.90)	12.30 (12.00, 12.80)	U = 4943.0	0.152
HCT (%)	40.70 (38.60, 44.70)	42.50 (40.20, 45.70)	U = 4411.0	0.007
RBC (×10^12^/L)	4.72 (4.34, 5.12)	4.84 (4.50, 5.21)	U = 4849.0	0.101
PCT (%)	0.26 ± 0.06	0.27 ± 0.06	t = −0.68	0.496
PLT (×10^9^/L)	262.77 ± 65.61	256.85 ± 55.57	t = 0.71	0.481
HGB (g/L)	139.00 (131.00, 153.00)	144.00 (135.00, 156.00)	U = 4540.0	0.014
**Derived markers**				
NLR	2.61 (1.83, 4.40)	1.50 (1.24, 1.80)	U = 8725.0	<0.001
PLR	143.15 (104.41, 184.96)	121.37 (89.56, 142.66)	U = 7016.0	<0.001
LMR	3.54 (2.74, 4.52)	5.02 (4.24, 5.93)	U = 2148.0	<0.001
SII	713.77 (437.42, 1062.90)	383.73 (300.54, 493.55)	U = 8163.0	<0.001
SIRI	1.32 (0.83, 2.32)	0.68 (0.46, 0.84)	U = 8226.0	<0.001
NPR	0.02 (0.01, 0.03)	0.01 (0.01, 0.02)	U = 8284.0	<0.001
ELR	0.04(0.02, 0.07)	0.06(0.03, 0.09)	U = 3686.0	<0.001
BLR	0.01(0.01, 0.02)	0.01(0.01, 0.02)	U = 6004.0	0.049

**Table 2:** Bootstrap-derived coefficients and odds ratios for the final L2-regularized logistic regression model.

Variables	β	OR	95% CI
age	−0.086	0.918	0.703–1.172
gender	0.143	1.154	0.881–1.502
NEUT	0.257	1.293	1.018–1.632
EO	−0.188	0.829	0.638–1.073
MPV	−0.426	0.653	0.479–0.877
HCT	−0.304	0.738	0.609–0.895
HGB	−0.282	0.754	0.620–0.908
NLR	0.36	1.433	1.178–1.732
LMR	−0.361	0.697	0.536–0.931
SII	0.234	1.264	1.040–1.568
SIRI	0.422	1.525	1.336–1.742
NPR	0.467	1.595	1.259–2.034

Note: β and 95% CI were estimated by bootstrap resampling (*n* = 1000, percentile method). Significance was defined as 95% CI excluding zero (i.e., OR 95% CI excluding 1).

**Table 3:** Performance metrics of models on the training set based on 10-fold cross-validation.

Model	*n*	AUC (Mean ± SD)	Accuracy	Sensitivity	Specificity	PPV	NPV	Brier Score
L2 Logistic	210	0.844 ± 0.099	0.814	0.745	0.886	0.866	0.784	0.156
Ridge Logistic	210	0.844 ± 0.099	0.814	0.745	0.886	0.866	0.784	0.156
Linear kernel SVM	210	0.841 ± 0.104	0.762	0.564	0.963	0.937	0.695	0.155

Note: *n* indicates the sample size of the training set. PPV, positive predictive value; NPV, negative predictive value.

**Table 4:** Performance metrics of models on the test set.

Model	*n*	AUC (95% CI)	Accuracy	Sensitivity	Specificity	PPV	NPV	Brier Score
L2 Logistic	90	0.859 (0.780–0.937)	0.767	0.711	0.822	0.800	0.740	0.158
Ridge Logistic	90	0.859 (0.780–0.937)	0.767	0.711	0.822	0.800	0.740	0.158
Linear kernel SVM	90	0.850 (0.769–0.931)	0.722	0.556	0.889	0.833	0.667	0.162

## Data Availability

The datasets are available on reasonable request to Xiaomei Fu (fuxiaomei202010@163.com), after approval from the Shanghai Mental Health Center, in accordance with local guidelines and regulations.

## Appendix A

Baseline characteristics of the full cohort were consistent with those observed in the training set.

**Table A1:** Baseline characteristics of the overall cohort.

Variables	SCZ (*n* = 150)	HC (*n* = 150)	*p*
Gender (female, %)	79 (52.7%)	79 (52.7%)	1.0000
Age (years)	36.00 (27.00, 47.75)	37.00 (28.00, 47.75)	0.3595
NEUT	4.96 (3.42, 7.10)	3.25 (2.63, 4.09)	<0.001
MONO	0.53 (0.42, 0.65)	0.43 (0.35, 0.53)	<0.001
BASO	0.03 (0.02, 0.04)	0.03 (0.02, 0.04)	0.7503
EO	0.07 (0.03, 0.13)	0.13 (0.08, 0.19)	<0.001
MCV	87.60 (85.20, 90.50)	88.60 (86.22, 90.90)	0.0364
MCHC	338.00 (332.00, 347.00)	337.50 (331.25, 343.00)	0.1572
MCH	29.85 (28.82, 30.80)	30.00 (29.10, 31.00)	0.3033
MPV	10.00 (9.40, 10.70)	10.30 (9.80, 10.90)	0.0018
LYMPH	1.77 (1.39, 2.29)	2.17 (1.86, 2.53)	<0.001
WBC	7.70 (6.05, 9.65)	6.06 (5.18, 7.16)	<0.001
RDW-CV	12.50 (12.00, 12.88)	12.30 (12.00, 12.80)	0.4195
HCT	40.60 (38.62, 45.25)	42.25 (39.82, 45.65)	0.0124
RBC	4.72 (4.31, 5.18)	4.79 (4.45, 5.20)	0.1869
PCT	0.26 (0.22, 0.31)	0.27 (0.23, 0.31)	0.4795
PLT	264.00 (223.25, 304.25)	261.50 (213.25, 298.00)	0.6667
HGB	138.50 (130.00, 153.75)	142.00 (135.00, 156.00)	0.0397
NLR	2.53 (1.87, 4.44)	1.46 (1.24, 1.81)	<0.001
PLR	141.95 (106.87, 189.68)	120.74 (90.21, 146.85)	<0.001
LMR	3.48 (2.52, 4.54)	5.04 (4.22, 6.18)	<0.001
SII	680.00 (456.16, 1105.20)	386.79 (298.49, 484.11)	<0.001
SIRI	1.31 (0.83, 2.32)	0.65 (0.46, 0.91)	<0.001
NPR	0.02 (0.01, 0.03)	0.01 (0.01, 0.02)	<0.001
ELR	0.04 (0.02, 0.07)	0.06 (0.04, 0.09)	<0.001
BLR	0.01 (0.01, 0.02)	0.01 (0.01, 0.02)	0.0171

Note: NEUT: neutrophil count, MONO: monocyte count, BASO: basophil count, EO: eosinophil count, MCV: mean corpuscular volume, MCHC: mean corpuscular hemoglobin concentration, MCH: mean corpuscular hemoglobin, MPV: mean platelet volume, LYMPH: lymphocyte count, WBC: white blood cell count, RDW-CV: red blood cell distribution width-coefficient of variation, HCT: hematocrit, RBC: red blood cell count, PCT: plateletcrit, PLT: platelet count, HGB: hemoglobin, NLR: neutrophil-to-lymphocyte ratio, PLR: platelet-to-lymphocyte ratio, LMR: lymphocyte-to-monocyte ratio, SII: systemic immune-inflammation index, SIRI: systemic inflammation response index, NPR: neutrophil-to-platelet ratio, ELR: eosinophil-to-lymphocyte ratio, BLR: basophil-to-lymphocyte ratio.
